# Hospital Diets in 1764

**Published:** 1906-04-21

**Authors:** 


### Hospital Diets in 1764.

It is not easy to get many details of hospital management in the middle of the eighteenth century. No excuse, therefore, is needed for reprinting the diets in use at the London Hospitals in 1764. They are taken from a small work called "The Modern Practice of the London Hospitals—viz., St. Bartholomew's, St. Thomas's, St. George's, and Guy's," published by W. Nicoll in St. Paul's Churchyard. The book is said to contain "exact copies of the receipts and a particular account of the different methods of cure at the different hospitals for the various diseases incident to the human body. Very proper for all physicians, surgeons, apothecaries, and particularly useful for all private families, especially those residing in the country." It begins with a table of diet.

Full Diet.

*Sunday and Thursday.*—Breakfast: A pint of water gruel. Dinner: Half a pound of boiled beef with greens. Supper: A pint of broth.

*Tuesday and Saturday.*—Breakfast: A pint of water gruel. Dinner: Half a pound of boiled mutton with greens. Supper: A pint of broth.

*Monday.*—Breakfast: A pint of milk pottage. Dinner: A pint of rice milk. Supper: Two ounces of cheese or butter.

*Wednesday.*—Breakfast: A pint of milk pottage. Dinner: Half a pound of boiled pudding. Supper: A pint of water gruel.

*Friday.*—Breakfast: A pint of milk pottage. Dinner: A pint of plumb broth. Supper: Two ounces of cheese or butter.

The patients upon full diet shall have one loaf of bread per day.

Three pints of small beer per day from Lady Day to Michaelmas. One quart per day from Michaelmas to Lady Day.

N.B.—The loaf of bread weighs fourteen ounces.

Low Diet.

*Sunday.*—Breakfast: A pint of water gruel. Dinner: Two ounces of roasted veal, with a slice of bread pudding. Supper: A pint of broth.

*Tuesday and Saturday.*—Breakfast: A pint of water gruel. Dinner: Two ounces of boiled mutton with greens and a pint of broth. Supper: A pint of broth.

*Monday.*—Breakfast: A pint of milk pottage. Dinner: A pint of rice milk. Supper: Two ounces of cheese or butter.

*Wednesday.*—Breakfast: A pint of milk pottage. Dinner: A slice of boiled pudding. Supper: A pint of water gruel.

*Thursday.*—Breakfast: A pint of water gruel. Dinner: Two ounces of roasted veal and a pint of rice milk. Supper: A pint of broth.

*Friday.*—Breakfast: A pint of milk pottage. Dinner: A pint of plumb broth. Supper: Two ounces of cheese or butter.

The patients upon low diet shall have one loaf of bread per day. One quart of beer per day from Lady Day to Michaelmas. One pint per day from Michaelmas to Lady Day.

Milk Diet.

*Sunday, Tuesday, Thursday, and Saturday.*—Breakfast: A pint of milk pottage or water gruel. Dinner: A pint of plumb pottage and four ounces of bread pudding. Supper: A pint of milk pottage or water gruel.

The patients upon milk diet shall have one loaf of bread per day. Three pints of drink per day, one pint whereof shall be milk and two water.

The patients upon fish diet shall have fish for dinner on Mondays, Wednesdays, and Fridays, if it can conveniently be had; if not, the low diet.

The patients upon dry diet shall have two ounces of butter or cheese for breakfast and the same for supper every day in the week, and the low diet for dinner, but without broth or rice.

Milk on Tuesdays, Thursdays, and Saturdays.

Bread and beer as those upon low diet.

The patients upon raisin diet shall have half a pound of raisins per day, as much bread as they can eat, a quart of decoct. guaiac. fort, and as much of the decoct. guaiac. tenue as they can drink.

The patients under salivation shall have one quart of milk per day and half a pound of mutton to be boiled for broth.

The following is the full diet at St. Bartholomew's Hospital in 1900 to compare with the foregoing.

Breakfast: Tea, $\frac{3}{4}$ pint; butter, $\frac{1}{2}$ ounce. Tea: Tea, $\frac{3}{4}$ pint; butter, $\frac{1}{2}$ ounce. During the remaining 24 hours: Meat six times a week, six ounces (dressed without bone). Fish once a week, ten ounces (dressed without bone). Potato, eight ounces, or when fresh vegetables are not too dear. Potato, six ounces and fresh vegetable four ounces. Pudding, four ounces. Milk, one pint. Bread eighteen ounces. The dietary, too, is not only more ample, but it is much more varied than the older one. On Sunday roast beef, Monday boiled mutton, Tuesday Irish stew, Wednesday roast mutton, Thursday boiled mutton, Friday fish, Saturday roast mutton. Puddings: Rice four times a week, sago once a week, suet twice a week (with golden syrup). Custard only when specially ordered. Dry arrowroot and cocoa in powder are to be kept in the wards for use when ordered. Beef tea, beef essence, mutton broth, and mutton essence to be supplied only when specially ordered.

